# Chronic Wasting Disease Agents in Nonhuman Primates

**DOI:** 10.3201/eid2005.130778

**Published:** 2014-05

**Authors:** Brent Race, Kimberly D. Meade-White, Katie Phillips, James Striebel, Richard Race, Bruce Chesebro

**Affiliations:** National Institute of Allergy and Infectious Diseases, National Institutes of Health, Hamilton, Montana, USA

**Keywords:** Chronic wasting disease, prion, transmissible spongiform encephalopathy, species barrier, transmission, non-human primate, squirrel monkey, cynomolgus macaque

## Abstract

Chronic wasting disease is a prion disease of cervids. Assessment of its zoonotic potential is critical. To evaluate primate susceptibility, we tested monkeys from 2 genera. We found that 100% of intracerebrally inoculated and 92% of orally inoculated squirrel monkeys were susceptible, but cynomolgus macaques were not, suggesting possible low risk for humans.

Chronic wasting disease (CWD) is a transmissible spongiform encephalopathy that can infect mammals in the family Cervidae, which includes deer, elk, and moose, among other species. Initially detected in a captive deer in 1967, the disease is now widespread in the United States, Canada, and South Korea (*1*). Because cervids are commonly consumed as food by humans and other mammals, the cross-species potential of the causal pathogen must be determined. CWD prions injected intracerebrally have infected agricultural animals and scavengers (*2*); however, transgenic mice that expressed human prion protein (PrP) were not susceptible (*3*–*6*) through this route. We previously analyzed the susceptibility of 2 genera of nonhuman primates to CWD agents by intracerebral and oral routes (*7*). The results showed a high attack rate in squirrel monkeys (*Saimiri sciureus*) inoculated intracerebrally but a low attack rate and long incubation periods by those exposed orally. In contrast, no cynomolgus macaques (*Macaca fascicularis*) showed clinical signs of transmissible spongiform encephalopathy (TSE) when exposed by either route. The long incubation periods observed in squirrel monkeys prompted us to observe the remaining monkeys for >4 additional years. Here we provide an update and report results of new experiments showing that squirrel monkey–adapted CWD (SM-CWD) has an accelerated incubation period on second passage.

## The Study

**Figure 1:**
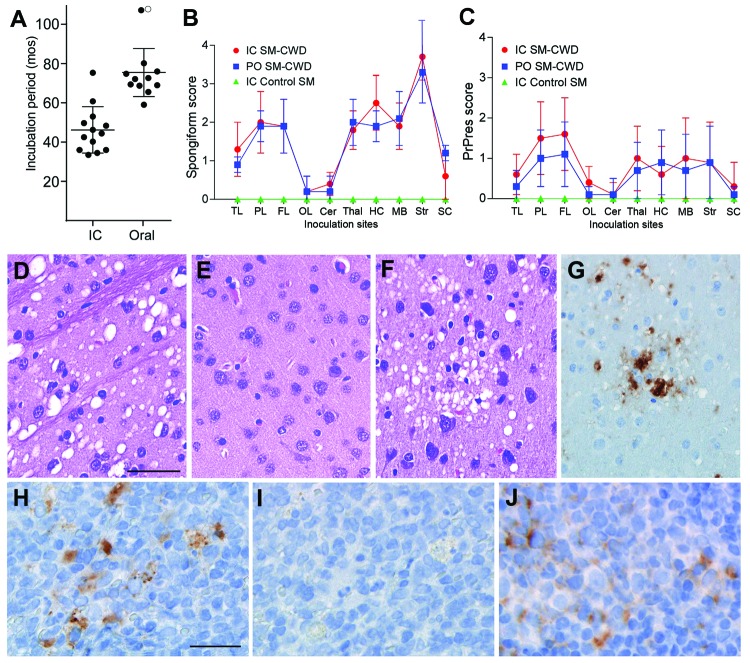
Incubation periods of chronic wasting disease (CWD) and neuropathologic features of CWD agent–infected squirrel monkeys. A) Incubation periods for squirrel monkeys infected with CWD agents by intracerebral (IC) or oral (PO) routes. Solid circles indicate euthanized squirrel monkeys (SM) that tested positive for prion disease. The open circle indicates 1 squirrel monkey that remained clinically normal at 108 months postinoculation (mpi). Lines indicate the mean and standard deviation within each group. B, C) Lesion profiles of CWD-agent–infected squirrel monkeys showing spongiform degeneration (B) and PrPres deposition (C) values in 10 gray matter regions of the brain. N values for each group are as follows: IC SM-CWD, 11; PO SM-CWD, 7; IC control SM, 1. TL, temporal lobe; PL, parietal lobe; FL, frontal lobe; OL, occipital lobe; Cer, cerebellum; Thal, thalamus; HC, hippocampus; MB, midbrain; Str, stiatum; SC, spinal cord. Error bars show the SD for each group. Panels D-G show brain from a squirrel monkey infected PO with CWD and euthanized at 69 months postinoculation. Panels D-F are stained with hematoxylin and eosin and show D) severe spongiform lesions in the striatum, E) lack of pathology in the occipital lobe, and F) pathology in the parietal lobe. Panels G, H, and J show immunohistochemical staining for PrPres by using anti-PrP antibody D13. G) Adjacent section to the region depicted in F shows the positive correlation of PrPres (brown) with spongiform degeneration. Panels H–J show lymphoid tissue from a squirrel monkey infected PO with CWD and euthanized at 80 mpi. H) PrPres (brown) staining in spleen and J) mesenteric lymph node. I) No primary antibody control of the region shown in H, demonstrating specificity of stain observed in H. The scale bar shown in D applies to panels D–G and represents 50 µm; the scale bar shown in H applies to H–I and represents 25 µm.

Squirrel monkeys were inoculated intracerebrally or orally with CWD inocula (*7*). We initially reported that 11/13 intracerebrally infected monkeys were euthanized at 41 months postinoculation (mpi) on average, and disease developed in 2/12 orally infected squirrel monkeys on average of 69 mpi (*7*). Disease developed in the 2 remaining intracerebrally infected squirrel monkeys at 61 and 75 mpi, respectively, changing the intracerebral attack rate to 100% (Figure 1, Table 1). Of the 10 remaining orally inoculated squirrel monkeys, disease developed in 9, bringing the overall oral attack rate to 92% and the average incubation period to 68 mpi (Figure 1, Table 1). Clinical signs were subtle; the most prominent finding was gradual weight loss (Table 1). A final diagnosis of CWD agent infection was made by using immunoblotting and immunohistochemical testing to determine accumulation of abnormal, disease-associated prion protein (PrPres) in brain tissue (Technical Appendix).

**Table 1:** Squirrel monkeys inoculated with CWD or squirrel monkey–adapted CWD agents*

Disease incidence†	Inoculum‡	Route of inoculation	Titer inoculated§	Incubation days, range, (avg)¶	Weight change range, % (avg,%)
13/13	MD-1,2,3 Elk-1,2,3 WTD-1,2	Intracerebral	1.3 × 10^5^-1.0 × 10^7^	33–75 (46)	−8 to −43 (−29.5)
11/12#	MD-1,3 Elk-1,2,3 WTD-1,2	Oral	9.6 x 10^7^-1.5 × 10^9^	59–107 (68)	−8 to −41 (−28)
2/2	SM-CWD	Intracerebral	NA	23–24 (23.5)	−8 to −21 (−14.5)
0/1	Normal elk	Intracerebral	NA	82 NS	0
0/1	Buffer control	Oral	NA	>108	−6
0/1	Normal elk	Oral	NA	123 NS	+7

*An early version of some of these data is shown in Tables 1, 2 of (*7*). Since that time more infected animals have been euthanized and the data have been updated. CWD, chronic wasting disease; MD, mule deer; WTD, white-tailed deer; NA, not applicable; NS, no clinical transmissible spongiform encephalopathy signs. †Number of monkeys in which prion disease developed/number inoculated. ‡Several different inocula were used for this study. Each individual animal was inoculated with 1 inoculum. Detailed descriptions can be found in (*7*). §Infectivity titers were determined by using endpoint dilution titer in transgenic deer PrP mice and are listed as 50% infectious dose/gram of brain. ¶The range of incubation periods observed is shown as months postinoculation followed with the average incubation period of the group in parentheses. Monkeys listed as NS did not show any clinical signs compatible with transmissible spongiform encephalopathy. #Three monkeys from this group are not included in this calculation because they were euthanized before 45 months postinoculation for reasons unrelated to transmissible spongiform encephalopathy disease. The sole remaining animal in this group appeared normal at 108 months postinoculation.

To compare the neuropathologic changes in intracerebrally and orally infected squirrel monkeys, we analyzed 10 brain regions for spongiform lesion severity and PrPres deposition (Figure 1, panels B, C). No statistically significant differences were noted between the 2 routes of infection (p<0.05). All squirrel monkeys studied had severe spongiform degeneration in the striatum (Figure 1, panel D) and little involvement in cerebellum and occipital lobes (Figure 1, panel E). Spongiform lesions in cortical gray matter were not consistent throughout the brain. Affected areas were commonly observed adjacent to normal regions, most frequently in the frontal, temporal, and parietal lobes (Figure 1, panel F). Except for the striatum, PrPres deposition was generally most prominent in areas that showed severe vacuolation. PrPres deposits appeared in 2 forms: dense punctate extracellular plaques (Figure 1, panel G) and less dense pericellular aggregates. The spleens of CWD agent–infected squirrel monkeys were positive for PrPres in 46% of intracerebrally infected and 60% of orally infected squirrel monkeys (Figure 1, panel H). At least 1 lymph node was positive in 30% of intracerebrally infected squirrel monkeys and in 40% of orally infected squirrel monkeys (Figure 1, panel J; Technical Appendix).

Of the squirrel monkeys under study, 3 *PRNP* genotypes were represented (*7*). In the group of orally infected squirrel monkeys, 3 had a unique heterozygous genotype that encoded either 4 or 5 octapeptide repeats. Two of these monkeys were the last orally infected monkeys to be euthanized because of clinical disease (80 and 107 mpi), and the third heterozygote was clinically normal at 108 mpi. Heterozygosity within the *PRNP* gene has been shown to delay or prevent prion disease (*8*) and may play a role in this study.

We inoculated cynomolgus macaques as another nonhuman primate model for cross-species transmission of CWD. Compared with squirrel monkeys, cynomolgus macaques are biologically closer to humans, and cynomolgus macaque PrP is more homologous to human PrP (*7*). Nine cynomolgus macaques were inoculated orally and 6 were inoculated intracerebrally with 1 of 3 CWD pools as described (*7*). Our first report included negative data from 1 cynomolgus macaque euthanized at 49 mpi (*7*). Since then, we have euthanized and screened 6 cynomolgus macaques for TSE (Table 2). No evidence of prion infection was detected by immunoblot and immunohistochemical methods (data not shown).

**Table 2:** Cynomolgus macaques inoculated with CWD or squirrel monkey–adapted CWD agents*

Disease incidence†	Inoculum‡	Route of inoculation	Titer inoculated§	Screening mpi¶	Current mpi
0/6	MD-1, Elk-1, WTD-1	Intracerebral	3.2 × 10^5^–2.5 × 10^6^	49, 79, 88, 94	124
0/8#	MD-1, Elk-1, WTD-1	Oral	2.5 × 10^8^–2 × 10^9^	97,106,106	124
0/2	SM-CWD	Intracerebral	NA	NA	72
0/1	Normal elk	Intracerebral	NA	96	NA

*An early version of some of these data are shown in Table 3 of (*7*). CWD, chronic wasting disease; mpi, months post-inoculation; MD, mule deer; WTD, white-tailed deer; SM, squirrel monkey; NA, not applicable. †Number of monkeys in which prion disease developed over number inoculated. ‡Several different inocula were used for this study. Each individual animal was inoculated with 1 inoculum. Detailed descriptions can be found in (*7*). §Infectivity titers were determined by using endpoint dilution titer in transgenic mice expressing deer prion protein (PrPres) and are listed as 50% infectious dose per gram of brain. ¶Several monkeys were euthanized during the course of the experiment for conditions unrelated to prion infection such as diabetes, neoplasia, hypocalcemia, and behavioral issues. Brain, spleen, and lymph nodes from these animals were screened for PrPres by using Western blot and immunohistochemical methods. No PrPres-positive tissues were detected. #One monkey from the original oral inoculation group was euthanized at 1 mpi because of a colonic torsion and has been removed from this group.

The lack of CWD transmission during >10 years suggests that a substantial species barrier exists between cervids and cynomolgus macaques. In most TSE animal models, PrPres can be detected by 1/3–1/2 of the known incubation periods. If we extrapolate this to the cynomolgus macaques in this study, negative test results at 9 years would suggest that the incubation period would be >18 years. Other prion studies of cynomolgus macaques reported clinical disease within 2–3 years after inoculation with variant Creutzfeldt-Jakob disease agents (*9*), 3 years after inoculation with bovine spongiform encephalopathy agents (*10*,*11*), and 5 years after inoculation with sporadic Creutzfeldt-Jakob disease agents (*9*,*12*). In contrast, our findings indicate that CWD is unlikely to develop in cynomolgus macaques.

The cause of susceptibility to CWD agents in squirrel monkeys and resistance to them in cynomolgus macaques is uncertain. *Prnp/PRNP* gene sequence variation has been linked to disease susceptibility (*8*), and differences in the *PRNP* genes of cynomolgus macaques and the genes of squirrel monkeys could play a major role. Comparison of *PRNP* sequences among cynomolgus macaques and squirrel monkeys showed differences exist at 5 codons (56, 100, 108, 159, and 182) (*7*). It is not clear which difference or combination of changes might confer protection to cynomolgus macaques, or if resistance is caused by other factors. Of the 5 codon differences described above, those of cynomolgus macaques and humans are identical at positions 56, 159, and 182.

**Figure 2:**
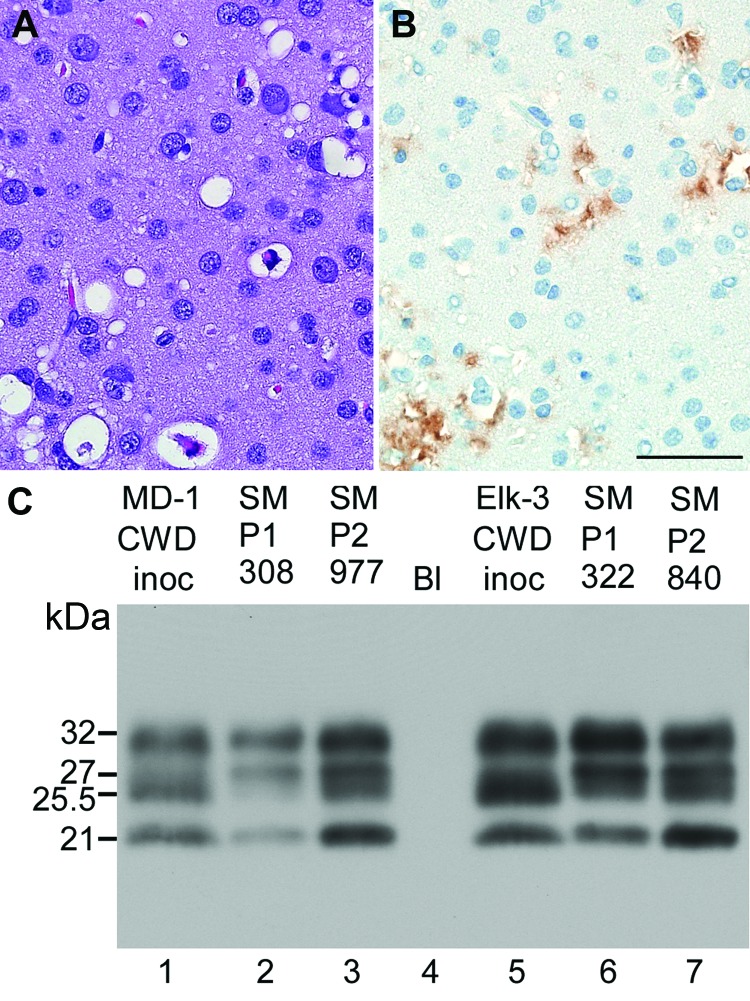
Neuropathologic features and immunoblot results of second-passage squirrel monkeys that had chronic wasting disease (CWD). Scale bar represents 50 µM and is applicable to panels A and B. Panels A and B show neuropathologic changes in the occipital lobe of SMP2-CWD monkey 977, which was euthanized at 24 months postinoculation. A) Hematoxylin and eosin staining show prominent spongiform changes. B) Immunohistochemical staining for disease-associated prion protein (PrPres) (brown) with anti-PrP antibody D13. C) Results of Western blot for PrPres in brain tissue of cervids and its respective first and second passage in squirrel monkeys. MD-1 was used to infect SM308, and SM308 was used to infect SM977. Lanes 1, 2, 5, and 6, 0.6 mg brain equivalents. Lanes 3 and 7, 0.36 mg brain equivalents to give similar signal intensities to the other samples. Lane 4, blank (Bl). Apparent molecular weights (in kDa) are provided on the left side of panel C. Immunoblot was probed with anti-PrP antibody L42. When comparing the 2 central bands, cervid CWD had a more intense band at 25.5 kDa; SM-CWD (nos. 308 and 322) and SM2-CWD (nos. 977 and 840) were more intense at 27 kDa.

Two SM-CWD brain samples were inoculated into squirrel monkeys and cynomolgus macaques to verify that SM-CWD was infectious, test for further adaptation, and to see if SM-CWD was infectious to a broader range of nonhuman primates. Two squirrel monkeys inoculated intracerebrally with SM-CWD brain homogenates (SMP2-CWD) were euthanized at 23–24 mpi (Table 1). These incubation periods decreased by >11 months compared with that of the donor squirrel monkey. Neurologic signs in the 2 SMP2-CWD were more pronounced than observed during the first passage; however, weight loss was reduced. Neuropathologic examination and Western blot for PrPres confirmed TSE in both squirrel monkeys. In contrast to SM-CWD infections, the SMP2-CWD-infected brains had spongiform lesions and PrPres deposition in the occipital lobe (Figure 2, panels A, B). Biochemical comparison of glycoform patterns among CWD, SM-CWD, and SMP2-CWD were made by using 3 different anti-PrP antibodies (L42, 6H4, and 3F4) (Technical Appendix). In all cases, SM2-CWD had a greater proportion of unglycosylated PrPres and a lower proportion of double glycosylated PrPres than did SM-CWD (Figure 2, panel C). The decreased time of manifestation of disease, differences in glycoform patterns, and distribution of PrPres in brain tissue suggested that the CWD agent was still adapting within the squirrel monkey. However, similar to CWD, SM-CWD had not caused prion disease in cynomolgus macaques by 72 mpi (Table 2).

## Conclusion

Our studies have shown that squirrel monkeys, but not cynomolgus macaques, were susceptible to CWD. Although these nonhuman primates are not exact models of human susceptibility, they support the data from transgenic mouse studies (*3*–*6*), in vitro experiments (*13*), and epidemiologic evidence (*14*,*15*) that suggest humans are at a low risk of contracting CWD. Nevertheless, it remains sensible to minimize exposure to tissues potentially contaminated with the CWD agent.

Technical Appendix Materials and methods for analysis of nonhuman primate tissues for chronic wasting disease in nonhuman primates.
