# Supplementary material for: Chronic Wasting Disease Agents in Nonhuman Primates
Source: Emerg Infect Dis. 2014 May;20(5):833–7. doi: 10.3201/eid2005.130778 (PMC4012792; doi:10.3201/eid2005.130778)
Supplement: Technical Appendix — Materials and methods for analysis of nonhuman primate tissues for chronic wasting disease in nonhuman primates. [file 13-0778-Techapp-s1.pdf]

Article DOI: 10.3201/eid2005.130778

Chronic Wasting Disease Agents in Nonhuman Primates

Technical appendix

Materials and Methods

Nonhuman Primates

Monkeys were housed individually at the Rocky Mountain Laboratories (RML) in an AAALAC International accredited facility and experimentation followed NIH RML Animal Care and Use Committee approved protocols. Further details were provided (*1*).

CWD, SM-CWD and SM2-CWD brain homogenates used for inoculation of primates

Eight pools of CWD-agent inocula were used in the primary passage studies in SM and CM. Infectivity levels and sources of each pool have been described in detail previously (*1*). For passage experiments using SM-CWD, 2 CWD agent positive squirrel monkeys (SM308 and SM322) were selected. SM308 was originally infected with MD-1 brain homogenate (BH) pool and euthanized at 36 mpi. SM322 was infected with Elk-3 BH pool and euthanized at 34 mpi. Both monkeys had readily detectable levels of PrPres in their brains and severe spongiform lesions. SM-CWD and second passage SM-CWD infected monkey (SMP2-CWD) BHs were prepared from frontal cortex as a 20% (w/v) solution in 0.01 M Tris HCl pH 7.4 and frozen at -80 C. The day of inoculation samples were thawed, vortexed, and sonicated for 1 minute. The 20% BH stock was then diluted to 10% in phosphate buffered balanced salt solution (PBBS) with 2% fetal bovine serum. Samples were cleared of large particulates by centrifuging at 425 x g for 5 minutes.

Inoculation of Monkeys

Methods for the initial oral (PO) and intracerebral (IC) CWD agent and control monkey inoculations were previously described (*1*). For inoculation of SM-CWD BH into SM the animals were anesthetized with ketamine HCL (20 mg/kg) intramuscularly. The hair on the skull was clipped, and the area over the left parietal lobe was scrubbed with betadine solution. A

dremel tool with a 1/16 inch diameter stainless steel sterile drill bit was used to create a small hole through the skull. Two hundred microliters of 10% SM-CWD BH was administered through a 30G ½ inch needle attached to a 1 ml syringe inserted through the predrilled hole into the parietal lobe of the brain. CM were anesthetized with ketamine HCL (10 mg/kg) and prepared as described above for the SM. Four hundred fifty microliters of 10% BH was inoculated using a 1 mL syringe and ½ inch long, 26 gauge needle. The approximate location of the inoculation was near the junction of the left temporal and parietal lobes.

Tissue Processing and Analysis for PrPres by Immunoblot

Tissues (brain, spleen or lymph nodes from infected or uninfected controls) were prepared as 20% (w/v) homogenates in ice cold 0.01M Tris pH 7.4 or sterile PBS pH 7.2 using either an Omni Tissue homogenizer with disposable hard tissue probe (Omni international, Marietta, GA) or Mini-Bead Beater (Biospec Products, Bartlesville, OK). Samples were vortexed and then sonicated 1 minute and frozen until analyzed. Preparation of samples for PrPres analysis was described previously (*2*). Briefly, mild detergents were added and samples were treated with 50 µg/ml of proteinase K (Roche cat#03115879001) for 45 minutes. The reaction was stopped by adding 2 µl of 0.1M phenylmethylsulfonyl fluoride and placed on ice for 5 minutes. An equal volume of 2X Laemmeli sample buffer (Biorad, Hercules, CA, USA) was added, and samples were boiled for 5 minutes. Additional un-treated samples from both infected and uninfected monkeys were used to observe PrPsen and control for non-specific reactivity.

Samples were run on a 16% Tris-glycine gel and proteins were transferred to Immun-Blot PVDF-P membranes (Bio-Rad, Hercules, CA) using an iBlot transfer system (Invitrogen, Carlsbad, CA) set to program 3 with a 7 minute transfer. PrP bands were detected with monoclonal antibody 3F4 diluted 1:3000 (residues 109-112) (*3*), D13 from cell culture supernatant diluted 1:100 (residues 96-106) (*4*), 6H4 diluted 1:10,000 (residues 144-152) (*5*) or L42 diluted 1:5000 (residues 145-163) (rBiopharm) (*6*). D13 does not react with CM PrP and was not used for this species. Membranes were incubated in primary antibody in TBS-T for 1 hour. Membranes were rinsed with TBS-T buffer and incubated with their appropriate horseradish peroxidase-conjugated secondary antibody (sheep anti-mouse IgG for 3F4, 6H4 and L42, sheep anti-human IgG for D13) at a 1:10,000 dilution in TBS-T for 45 minutes. Bands were detected using enhanced chemiluminescence (ECL) substrate as recommended by the manufacture (Thermo Scientific, Rockford, Illinois). Densitometry studies on the glycoform

ratios were done using a Gel Doc XR and Quantity One software (Bio-Rad). Each sample was analyzed 2-4 times with three different anti-PrP antibodies (L42, 3F4, and 6H4). Results for each monkey with an individual antibody were averaged.

Histology & Immunohistochemistry

Tissues were placed in 3.7 % phosphate-buffered formalin for 3-5 days before dehydration and embedding in paraffin. Serial 4 µm sections were cut using a standard Leica microtome, placed on positively charged glass slides and dried overnight at 56º C. Sections were stained by standard hematoxylin and eosin methods (H&E). Immunohistochemical staining was performed using Ventana automated Nexus stainer (Ventana, Tucson, AZ). Slides were de-paraffinized and rehydrated to Tris-HCl buffer, pH 7.5. Anti-PrP staining was done using anti-PrP antibodies D13 and 3F4 as previously described (*4,7*). Uninfected SM and CM were used to observed PrPsen staining and potential non-specific reactivity. In addition, tissues were stained with the primary antibody omitted to observe non-specific staining due to the secondary antibody.

Brain and Lymphoid Pathology

Each squirrel monkey brain was scored for the degree of spongiform degeneration (H&E slides) and PrPres deposition (antibody D13 slides) in 10 brain regions. Brains were randomized and scored blind by one observer. A scale of 0-4 was used for each parameter. For spongiform degeneration the following method was used: 0, no vacuoles; 1, few vacuoles widely and unevenly distributed; 2, few vacuoles evenly distributed; 3, moderate numbers of vacuoles evenly distributed; and 4, many vacuoles with some confluences. PrPres deposition was scored in the same regions as follows: 0, no deposition; 1, 1–20% of area has visible PrPres plaques; 2, 20–40% of area covered with plaques; 3, 41–60% of area covered with plaques; and 4, >61% of area covered with plaques. Group mean scores were analyzed statistically using Prism 5.0c software (GraphPad Software) and plotted with their standard deviations to show representative lesion profiles.

Spleens and lymph nodes (LN) stained with D13 or 3F4 were also observed for PrPres deposition. No primary controls were used for each tissue to exclude non-specific staining from analysis. In addition, LN and spleens from uninfected squirrel monkeys were used as negative control tissue. A total of 78 lymph nodes (2-9 lymph nodes/monkey) and two sagittal sections of

spleen were observed. Lymph nodes from various anatomic locations were used including (47 mesenteric, 11 cervical, 11 axillary, 4 mandibular, 2 inguinal, 2 brachial and 1 ileocecal lymph node).

References

1. Race B, Meade-White KD, Miller MW, Barbian KD, Rubenstein R, LaFauci G et al. Susceptibilities of nonhuman primates to chronic wasting disease. Emerg Infect Dis. 2009; 15(9):1366-1376.

2. Meade-White K, Race B, Trifilo M, Bossers A, Favara C, Lacasse R et al. Resistance to chronic wasting disease in transgenic mice expressing a naturally occurring allelic variant of deer prion protein. J Virol. 2007; 81(9):4533-4539.

3. Kascsak RJ, Rubenstein R, Merz PA, Tonna-DeMasi M, Fersko R, Carp RI et al. Mouse polyclonal and monoclonal antibody to scrapie-associated fibril proteins. J Virol. 1987; 61(12):3688-3693.

4. Tribouillard-Tanvier D, Race B, Striebel JF, Carroll JA, Phillips K, Chesebro B. Early cytokine elevation, PrPres deposition, and gliosis in mouse scrapie: no effect on disease by deletion of cytokine genes IL-12p40 and IL-12p35. J Virol. 2012; 86(19):10377-10383.

5. Korth C, Stierli B, Streit P, Moser M, Schaller O, Fischer R et al. Prion (PrPSc)-specific epitope defined by a monoclonal antibody. Nature. 1997; 390(6655):74-77.

6. Vorberg I, Buschmann A, Harmeyer S, Saalmuller A, Pfaff E, Groschup MH. A novel epitope for the specific detection of exogenous prion proteins in transgenic mice and transfected murine cell lines. Virology. 1999; 255(1):26-31.

7. Klingeborn M, Race B, Meade-White KD, Chesebro B. Lower specific infectivity of protease-resistant prion protein generated in cell-free reactions. Proc Natl Acad Sci U S A. 2011; 108(48):E1244-E1253.
